# Involvement of INS15 in the development and pathogenicity of the zoonotic pathogen *Cryptosporidium parvum*

**DOI:** 10.1371/journal.pntd.0012569

**Published:** 2024-10-03

**Authors:** Wei He, Hao Cui, Na Li, Yaqiong Guo, Songrong Zeng, Yaoyu Feng, Lihua Xiao, Rui Xu

**Affiliations:** 1 State Key Laboratory for Animal Disease Control and Prevention, South China Agricultural University, Guangzhou, China; 2 School of Biology and Agriculture, Shaoguan University, Shaoguan, China; Cornell University, UNITED STATES OF AMERICA

## Abstract

**Background:**

*Cryptosporidium parvum* is a common protozoan pathogen responsible for moderate to severe diarrhea in humans and animals. The *C*. *parvum* genome contains 22 genes encoding insulinase-like M16 proteases (INS) with diverse structures and sequences, suggesting that members of the protein family may have distinct biological functions in the life cycle of parasites. Here, we investigated the role of INS15 and INS16, two proteases encoded by neighboring genes with high sequence identity, in the growth and development of *C*. *parvum in vivo* and *in vitro*.

**Methodology/Principal findings:**

*INS15* and *INS16* genes were tagged and knocked out using CRISPR/Cas9 technology in *C*. *parvum* IIdA20G1-HLJ isolate. The expression of INS15 and INS16 was determined by immunofluorescence analysis and immunoelectron microscopy. The effect of depletion of *INS15* and *INS16* on parasite growth and pathogenicity were assessed on HCT-8 cells and in interferon-γ knockout mice. Endogenous tagging showed that INS15 and INS16 expressed in the oocyst, trophozoite, meront and female gametes. INS15 also expressed in male gamonts, while INS16 was not detected in the male gamonts. Although depletion of the *INS15 or INS16* gene affected late development of *C*. *parvum in vitro*, only depletion of *INS15* significantly reduced parasite burden in infected mice. Mice infected with the *INS15*-depleted strain had reduced clinical signs, body weight, intestinal villus length to crypt height ratio, and survival time compared to infected with the tagging mutant.

**Conclusions/Significance:**

The results of this study indicate that INS15 is mainly involved in the late development of *C*. *parvum*. Depletion of this gene attenuates the pathogenicity of this important zoonotic parasite.

## Author summary

*Cryptosporidium parvum* is a common protozoan pathogen responsible for moderate to severe diarrhea in young children and animals and there is currently no effective treatment to prevent the many deaths it causes each year. Previous studies showed that *C*. *parvum* genome encodes 22 insulinase-like M16 proteases (INSs) with diverse structures and sequences, suggesting that members of this protein family may have distinct biological functions in the life cycle of parasites. Our work characterizes two INSs, INS15 and INS16, which are encoded by neighboring genes with high sequence identity. We used CRISPR/Cas9 genome editing to endogenous tagged INS15 and INS16 and showed that expression patterns of INS15 and INS16 were different, with INS16 was not detected in the male gamonts. Genetic ablation of INS15 or INS16 affected late development of *C*. *parvum in vitro*, while only depletion of INS15 significantly reduced parasite burden in infected interferon-γ knockout mice. The INS15-depleted strain resulted on a reduction of clinical signs and an extension of survival time in infected mice compared to the INS15-tagged strain. These findings demonstrate that the depletion of INS15 attenuates the pathogenicity of this important zoonotic parasite.

## Introduction

The intracellular pathogens *Cryptosporidium* spp. are apicomplexan parasites and leading causes of enteric disease in humans and animals [1]. *Cryptosporidium* is the second most common cause of diarrhea in children in developing countries, leading to recurrent or persistent infections and even death [1]. In addition, recently several outbreaks of cryptosporidiosis in newborn calves occurred in China, resulting in high mortality [2–4]. Nitazoxanide is the only FDA-approved drug for treatment of cryptosporidiosis, however, it is ineffective in malnourished and immunocompromised patients [5]. There is only one commercially approved effective *Cryptosporidium* vaccine that protects newborn calves from severe diarrhea [6]. The development of effective drugs and vaccines is hampered by a lack of understanding of the biology of *Cryptosporidium* spp. The recent development of CRISPR/Cas9 technology for genetic modification of *Cryptosporidium* has greatly facilitated studies of the numerous hypothetical proteins in the genome through gene tagging and depletion [7,8].

Secreted proteases and protein kinases in apicomplexan secretory organelles are known to be involved in processing invasion-related complexes or modifying host cell activities, thus playing important roles in invasion [9]. Insulinase-like proteases (INS), which belong to the M16 family of metalloproteases, are members of this large group of enzymes. A classical M16 protease normally contains four domains: an active domain, characterized by the presence of an inverted zinc-binding motif, and three inactive catalytic domains [10]. The function of M16 metalloproteases has been described in apicomplexans. Falcilysin in *Plasmodium falciparum* has been identified as a key component of the catabolic process and plays an important role in hemoglobin degradation [11]. Toxolysin 1 and toxolysin 4 in *Toxoplasma gondii* are found in rhoptries and micronemes, respectively, which play key roles in host cell interactions [12,13].

The small *C*. *parvum* genome contains 22 genes encoding INS, which may play important roles in the invasion and development of the pathogen. In addition, INS could be potential targets for the development of treatments for cryptosporidiosis. In previous studies, several INS proteins such as INS5, INS15, INS16, INS-20-19, INS21 and INS23 were shown to be potentially involved in host cell infection and development by antibody neutralization [14–18]. In addition, depletion of the *INS1* gene reduced the formation of macrogamonts and oocysts [19]. However, genetic tagging and depletion have not been used to characterize other INSs of *C*. *parvum*.

In the present study, we performed characterizations of INS15 and INS16 using CRISPR/Cas9 technology. Compared to other INS members in *C*. *parvum*, that mostly have only two of the four domains of classical M16 metalloproteases, INS15 is a classical M16A family member with four functional domains. In contrast, INS16 has three functional domains, lacking an M16 inactive domain. We define that INS15 is associated with pathogenicity *in vivo* and *in vitro*, whereas deletion of INS16 does not affect *Cryptosporidium* growth and pathogenicity.

## Material and methods

### Ethics statement and maintenance of mice

All animal work was performed with the approval of the Institutional Animal Care and Use Committee, South China Agricultural University (No. 2021c073). Interferon-γ knockout (GKO) mouse of the C57BL/6-J strain was purchased from the Institute of Laboratory Animals Science, Chinse Academy of Medical Sciences and bred in-house at the Laboratory Animal Center of South China Agricultural University. When necessary, female and male mice (3–5 weeks old) were randomly assigned to groups in a 1:1 ratio based on body weight for the generation and passage of transgenic *C*. *parvum* strains or to compare oocyst shedding of gene-tagged and depleted *C*. *parvum* strains. Mice were maintained in micro-isolators (Tecniplast, Inc., Shanghai, China) and fed sterile chow and filtered tap water. During infection experiments, mice were inoculated with transgenic strains and received a concentration of 16 g/L paromomycin sulfate salt (Sigma-Aldrich, St. Louis, MO, USA) in drinking water. They were monitored for body weight, coat condition, posture, and activity. Mice that lost more than 20% of their body weight or appeared nonambulatory were euthanized.

### Parasites and host cells

*Cryptosporidium parvum* IIdA20G1-HLJ isolate was obtained from a dairy farm in Heilongjiang, China [2] and maintained in newborn male Holstein calves from a *C*. *parvum*-free farm as previously described [20]. Prior to infection, purified oocysts were treated with 10% Clorox (0.52% sodium hypochlorite) on ice for 10 min and washed three times with PBS. The treated oocysts were resuspended in 1% BSA-PBS (wt/vol) and stored at 4°C. To obtain free sporozoites, bleached oocysts were excysted by incubation at 37°C for 60 min in the presence of 0.75% taurocholic acid (Sigma-Aldrich).

Human ileocecal adenocarcinoma HCT-8 cells were obtained from the Shanghai Branch of the Chinese Academy of Sciences. For parasite infection, HCT-8 cells were seeded into 24-well cell culture plates and cultured in RPMI 1640 medium containing 10% fetal bovine serum (FBS) at 37°C until ∼80% confluence. Cells were infected with 2 × 10^5^ bleached oocysts per well and cultured in RPMI 1640 medium containing 2% FBS. After 3 hours of infection, uninvaded parasites were washed from the culture with PBS. Fresh RPMI 1640 medium supplemented with 2% FBS was added to the culture, which was maintained for the duration of the assay.

### Bioinformatic analysis of INS15 and INS16

Amino acid sequences of *C*. *parvum* INS were downloaded from CryptoDB (https://cryptodb.org/cryptodb/app). Functional domains in the predicted amino acid sequences were searched using SMART (Simple Modular Architecture Research Tool)[21]. Sequences were aligned using clustalx1.81 and manually adjusted using BioEdit v7.2.5.0. Similarity analysis was performed using SimPlot v3.5.1.

### Primers

All primers were synthesized by Sangon Biotech (Sangon, Shanghai, China) and were listed in S1 Table in the supplemental material.

### Construction of CRSPR/Cas9 plasmids for gene depletion

To increase the efficiency of genetic manipulation of *INS15* and *INS16*, we used double-guide RNA (dgRNA) strategy to delete the target genes. To obtain the dgRNA plasmid for *INS15* and *INS16* depletion, we designed the *INS15* and *INS16* knockout (*Δins15* and *Δins16*) dgRNA using the eukaryotic pathogen CRISPR guide RNA/DNA design tool (http://grna.ctegd.uga.edu) in an analysis of the *C*. *parvum* IIdA20G1-HLJ genome to avoid off-target effects. The dgRNA of *INS15* targeted the region 207 bp upstream of the stop codon and 319 bp downstream of the start codon in the *INS15* (cdg3_4260). The dgRNA of *INS16* targeted the region 74 bp upstream of the stop codon and 128 bp upstream of the start codon in *INS16* (cdg3_4270). Plasmids pACT1::Cas9-U6::sgINS15-1-U6::sg INS15-2 and pACT1::Cas9-U6::sgINS16-1-U6::sg INS16-2 were generated by adding the two sgRNA targeting into the linear Cas9 plasmid via Gibson assembly as described [19,22,23]. To generate *Δins15* and *Δ ins16*, the Nluc-P2A-neo fragment and the plasmid backbone were amplified from the plasmid INS1-mCherry-Nluc-P2A-neo-INS1 [19], and homologous arms (900 bp upstream and 899 bp downstream of *INS15*, 908 bp upstream and 875 bp downstream of *INS16*) were amplified from *C*. *parvum* genomic DNA. Then, the INS15-Nluc-P2A-neo-INS15 and INS16-Nluc-P2A-neo-INS16 targeting plasmids were assembled in four fragments using Gibson assembly.

### Construction of CRSPR/Cas9 plasmids for epitope tagging

For tagging of genes, a specific single guide RNA (sgRNA) plasmid for the genetic tagging of INS15 and INS16 were generated as described above, respectively. To generate the tagging plasmid, a fragment of *INS15* and *INS16* C-terminus (697 and 384 bp) with the mutant protospacer adjacent motif and 3’ UTR of *INS15 and INS16* (899 and 322 bp) were amplified from *C*. *parvum* genomic DNA. The 3HA-Nluc-P2A-neo reporter and the backbone of pUC19 was amplified from pINS1-3HA-Nluc-P2A-neo[19]. The tagging plasmids pINS15-3HA-Nluc-P2A-neo and pINS16-3HA-Nluc-P2A-neo were assembled in four fragments by Gibson assembly.

### Selection and amplification of transgenic parasites

Sporozoites released from 1 × 10^7^ oocysts were electroporated with the respective 50 μg CRISPR/Cas9 plasmid and 50 μg targeting plasmid on an AMAXA 4D-Nucleofector system (Lonza). Then, the mixtures containing transfected sporozoites were diluted with 200 μL sterile PBS and administered to three GKO mice by oral gavage as described previously [19]. Transgenic parasites were selected from GKO mice with 16 g/L paromomycin provided in drinking water. Fecal pellets were collected starting at day 5 post-infection and stored at 4°C for luciferase assay or purification. Oocysts were purified from the fecal pellets using discontinuous sucrose and cesium chloride gradients as described previously [24].

### PCR identification of transgenic parasites

Genomic DNA was extracted from 100 mg of luciferase positive fecal material using the Fast DNA Spin Kit for Soil (MP Biomedical, Santa Ana, CA, USA). To confirm the insertion of the target sequence into the *INS15* and *INS16* gene loci, PCR was performed on the extracted fecal genomic DNA using Phanta Max Super-Fidelity DNA Polymerase (Vazyme, Nanjin, China). The primers used for confirming the correct 5’ and 3’ integration after homologous recombination of the transgenic strains are listed in S1 Table.

### Luciferase assay to measure parasite burden

Fecal luciferase activity was measured using the Nano-Glo Luciferase Assay (Promega, Madison, WI, USA) as described [22]. Briefly, weighed fecal pellets, ten 3-mm glass beads (ThermoFisher, Waltham, MA, USA), and 1 mL fecal lysis buffer (50 mM Tris-HCI pH 7.6, 2 mM DTT, 2 mM EDTA, 10% glycerol, and 1% Triton X-100) in a microcentrifuge tube were vortexed for 60 s in a FastPrep instrument (Qbiogene, Carlsbad, CA, USA). After centrifugation at 15,000 × g for 3 min, 50 μL of supernatant and 50 μL of Nano-Glo Luciferase Substrate Mix diluted 1:50 in Nano-Glo Luciferase Substrate with (Promega) were added to each well of a 96-well plate (Costar, Cambridge, MA, USA). The plate was incubated at room temperature for 3 min and luminescence was measured using a BioTek Synergy H1 Hybrid plate reader (BioTek, Winooski, VT, USA).

### Indirect immunofluorescence assay (IFA)

To assess expression of INS15 and INS16 in oocysts and intracellular stages of *C*. *parvum*, transgenic oocysts were resuspended in PBS and dried on poly-L-lysin-treated microscope slides (Waterborne, New Orleans, LA, USA), while HCT-8 cells infected with bleached oocysts were grown on coverslips for 24 and 48 h. The slides and coverslips were fixed with methanol for 15 min and permeabilized with 0.5% Triton X-100 in PBS for 30 min. After blocking with 1% BSA in PBS for 1 h at room temperature, the slides and coverslips were probed with rabbit anti-HA antibody diluted 1:800 (Cell Signaling Technology, Danvers, MA, USA) in 1% BSA-PBS for overnight at 4°C, followed by Alexa Fluor 488-conjugated goat anti-rabbit IgG diluted 1:400 (Cell Signaling Technology) and Sporo-Glo diluted 1:20 (Waterborne) for 1 h at room temperature, and the nuclei were stained with 4’, 6-diamidino-2-phenylindole (DAPI, Roche, Basel, Switzerland) for 10 min at room temperature. After three washes with PBS, the slides and coverslips were mounted with anti-fade mounting medium (Boster, Wuhan, China) and examined on a BX53 microscope (Olympus, Tokyo, Japan). Images were analyzed by ImageJ2 (https://imagej.nih.gov/ij/) or Zeiss software (ZEN 2).

### Assessment of *in vitro* development of transgenic parasites

HCT-8 cells were seeded on 24-well plates and grown to 80% confluence. Bleach-treated transgenic oocysts (1 × 10^4^ oocysts per well) were used to infect the HCT-8 monolayer for 3, 12, 24, 36 and 48 h. Cultures infected with transgenic oocysts were washed twice with PBS for removing uninvaded parasites and replenished with fresh medium containing 2% FBS at 3-hour post infection. At various time points, monolayers were fixed with methanol and stained with Sporo-Glo antibodies diluted 1:20 (Waterborne). Coverslips were examined and images were acquired on a BX53 microscope with capturing 30 random microscope fields per coverslip as previously described [25]. Three independent experiments were performed to determine the effect of gene deletion on parasite growth and proliferation during the intracellular period. The parasite numbers were analyzed using ImageJ.

### Assessment of pathogenicity of *Δins15* and *Δins16* strains *in vivo*

To assess the effect of INS15 and INS16 depletion on pathogenicity, GKO mice were infected with INS15-HA, *Δins15*, INS16-HA or *Δins16* transgenic oocysts (1 × 10^3^ oocysts/mouse) by oral gavage, six mice for each transgenic strain (n = 6 per group) and paromomycin drinking water was used after infection. Fecal pellets were collected every two days starting 2 days post-infection. Oocyst shedding in the mice was evaluated by luciferase assay as described above. The body weight of mice was recorded before infection and every two days after infection.

### Light and electron microscopy analyses

GKO mice were orally gavaged with 1 × 10^3^ transgenic oocysts (n = 2 per group). After 12 days post-infection, mice were euthanized, and ileal tissues were harvested for conventional microscopic examination of hematoxylin and eosin-stained sections. The remaining ileal tissue was fixed in 4% paraformaldehyde, dehydrated through various gradients of ethanol, and polymerized in LR White resin. Ultrathin sections were blocked and incubated with rabbit anti-HA diluted 1:20 (Cell Signaling Technology) and goat anti-rabbit IgG conjugated with 10 nm colloidal gold diluted 1:20 (Sigma-Aldrich) as primary and secondary antibodies, respectively. Then, sections were examined by immunoelectron microscopy on Talos L120C (ThermoFisher Scientific) as described [19].

### Statistical analysis

All values are presented as mean ± SD from at least three biological replicates. Statistical analysis was conducted in GraphPad Prism 9.5.0. Information on the specific statistical tests and *p* values are given in the legends for each figure. No data were excluded from analyses, *p* values < 0.05 were considered significant.

## Results

### INS15 and INS16 contain multiple domains and different active motif

**Fig 1:**
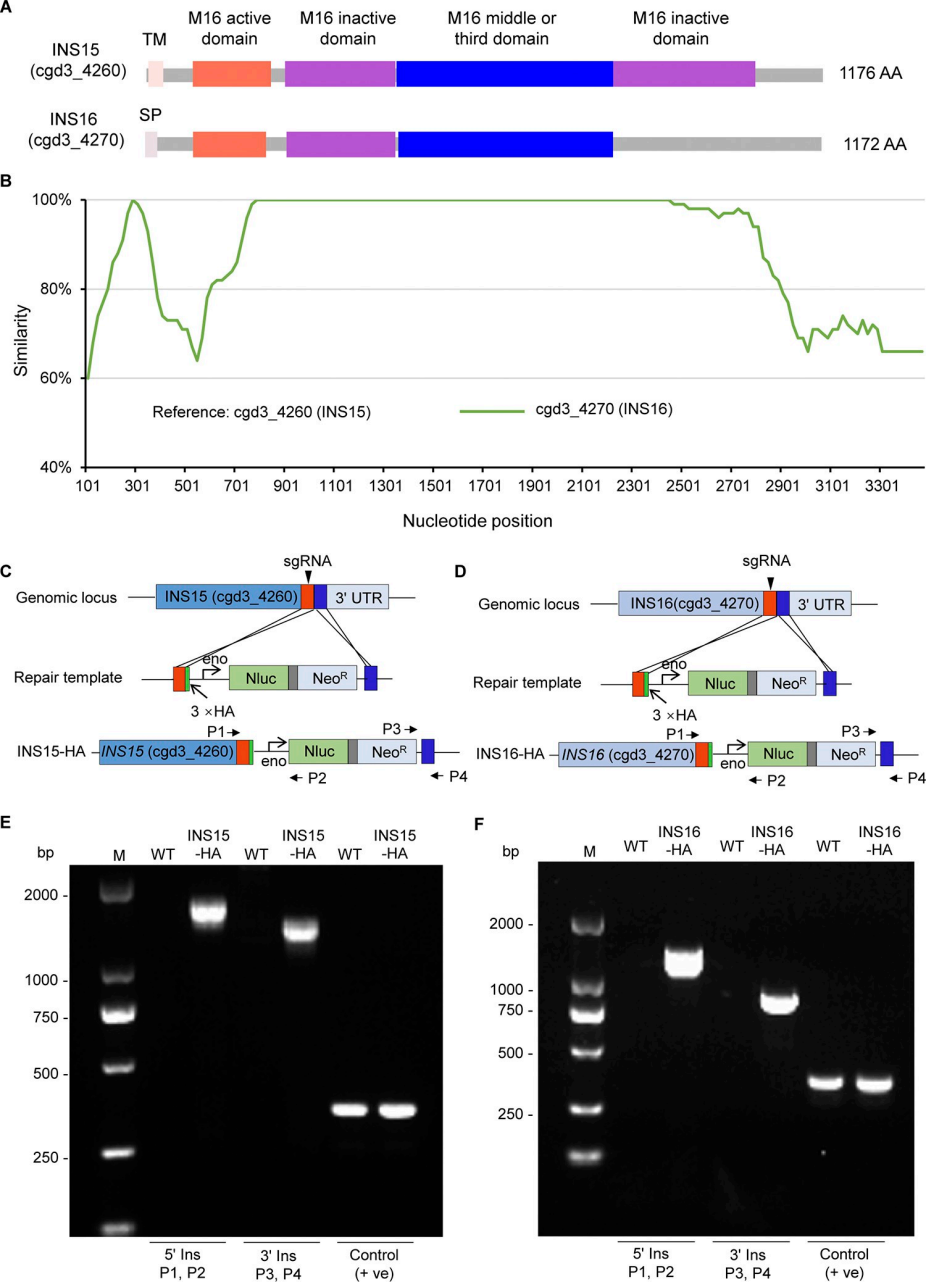
Sequence characteristics of *INS15* and *INS16* in *C*. *parvum*. (A) Domain architecture of INS15 and INS16. SP, signal peptide. TM, transmembrane domain (B) Similarity plot of the full-length gene sequences of *INS15* and *INS16*. *INS15* has a high sequence identity to *INS16* in most regions of the gene. (C-D) Schematic showing the tagging strategy of *INS15* and *INS16*. The top line shows the native locus of *INS15* or *INS16*, the middle line shows the repair template, and the bottom line shows the modified locus. sgRNA, single guide RNA; 3HA, the triple hemagglutinin epitope tag; Nluc, nano-luciferase; Neo^R^, neomycin resistance marker; Eno, enolase promoter. P1, P2, P3, and P4 are primers used to verify genomic integration events. (E-F) Diagnosis PCR of the INS15-HA or INS-16 locus. Fecal genomic DNA from wild-type (WT) and tagging (INS15-HA, INS16-HA) parasites were used as template, and primers for checking 5’ insertion (5’ Ins), 3’ insertion (3’ Ins), and the 3’ UTR sequence of INS16 as a positive control (+ve) are indicated.

*Cryptosporidium parvum* contains 22 INS proteins, of which INS15 (cgd3_4260) and INS16 (cgd3_4270) are located in the subtelomeric region of chromosome 3. Phylogenetic analysis showed that INS15 and INS16 were most similar to each other [19], however, the functions of INS15 and INS16 haven’t been explored yet. INS15 has four domains, an active domain containing the zinc-binding motif HLIEH and three inactive domains, consistent with the classical M16A clan. While INS16 also contains an active domain with the loss of an inactive domain at the C-terminus in contrast to classical INS (Fig 1A). To better understanding of their relationships, we then compared the similarity of INS15 and INS16 using SimPlot. The result showed that their differences were mainly in the active domain at the N-terminus (101–761 bp) and the inactive region at the C-terminus of the proteins (2821–3461 bp) (Fig 1B).

### Endogenous tagging reveals stage-specific expression of INS15 and INS16

To understand the localization of INS15 and INS16, we generated stable transgenic strains with a triple hemagglutinin (3×HA) epitope tag at the C-terminus of INS15 and INS16 proteins and the Nluc-Neo^R^ selection cassette after the endogenous locus (Fig 1C and 1D). Transgenic strains were generated and selected from GKO mice (n = 3), and PCR analysis of fecal genomic DNA confirmed that the 3×HA epitope tag and selection cassette were corrected inserted into the C-terminus of *INS15* and *INS16* genes (Fig 1E and 1F).

**Fig 2:**
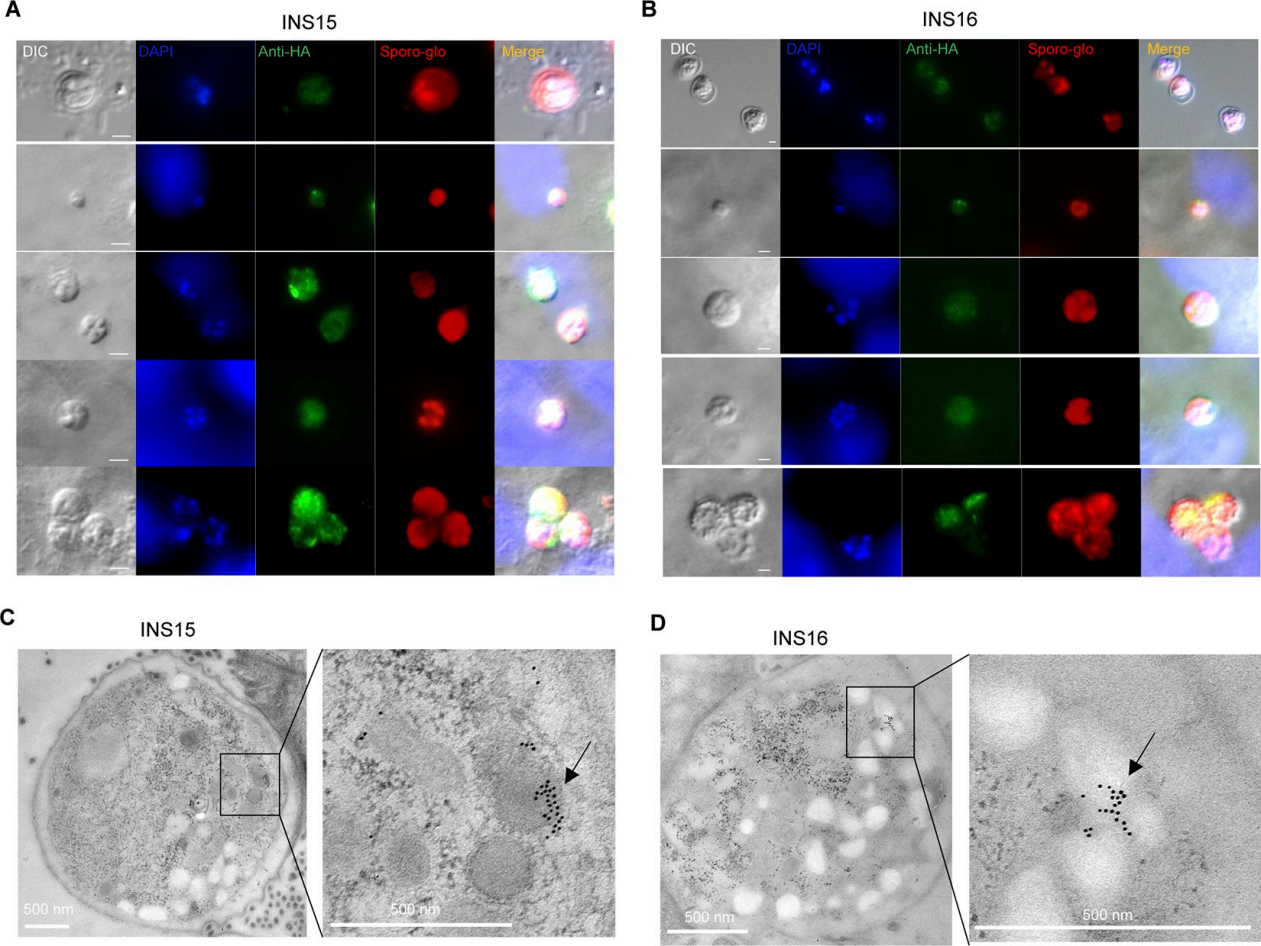
Localization of *C*. *parvum* INS15 and INS16 *in vitro*. (A-B) Immunofluorescence analysis of transgenic INS15-HA and INS16-HA parasites. For extracellular straining, INS15-HA or INS16-HA oocysts were fixed on the slides. For intracellular staining, HCT-8 cells were infected with transgenic oocysts and fixed at 24 hours post-infection (hpi) or 48 hpi. Then, samples were stained with rabbit anti-HA antibodies (green) and Sporo-glo antibodies that recognizes *C*. *parvum* (red) and DAPI (blue). Scale bars = 2 μm. (C-D) Immunoelectron microscopy of INS15-HA and INS16-HA parasites at female gametophyte stage. GKO mice were infected with INS15-HA or INS16-HA oocysts, and ileal tissues were fixed and stained with rabbit anti-HA followed by 10-nm colloidal gold goat anti-rabbit IgG. Black arrows indicate distribution of gold particles. Scale bars = 500 nm.

To visualize the stage-specific expression and localization of INS15 and INS16, we performed immunofluorescence analysis of INS15-HA and INS16-HA strains. INS15 was found to be expressed in oocysts and intracellular stages of *C*. *parvum* (Fig 2A). In contrast, INS16 was not expressed in the male gamont stage of *Cryptosporidium* development (Fig 2B). To determine subcellular localization, transgenic parasites were examined by immunoelectron microscopy. Anti-HA golden particles were observed in large vesicles of female gametes of the INS15-HA strain, while golden particles were observed only in bright vesicles of female gametes of the INS16-HA strain (Fig 2C and 2D). We attempted to observe the subcellular localization of INS15 and INS16, but some gold particles were scattered during other developmental stages, making it impossible to determine their expression in subcellular organelles (S1 Fig). These results suggest that INS15 and INS16 are differentially expressed in male gamonts, and they may play an important role mainly in the sexual reproduction stage of *C*. *parvum*.

### INS15 and INS16 are dispensable for *C*. *parvum* survival

**Fig 3:**
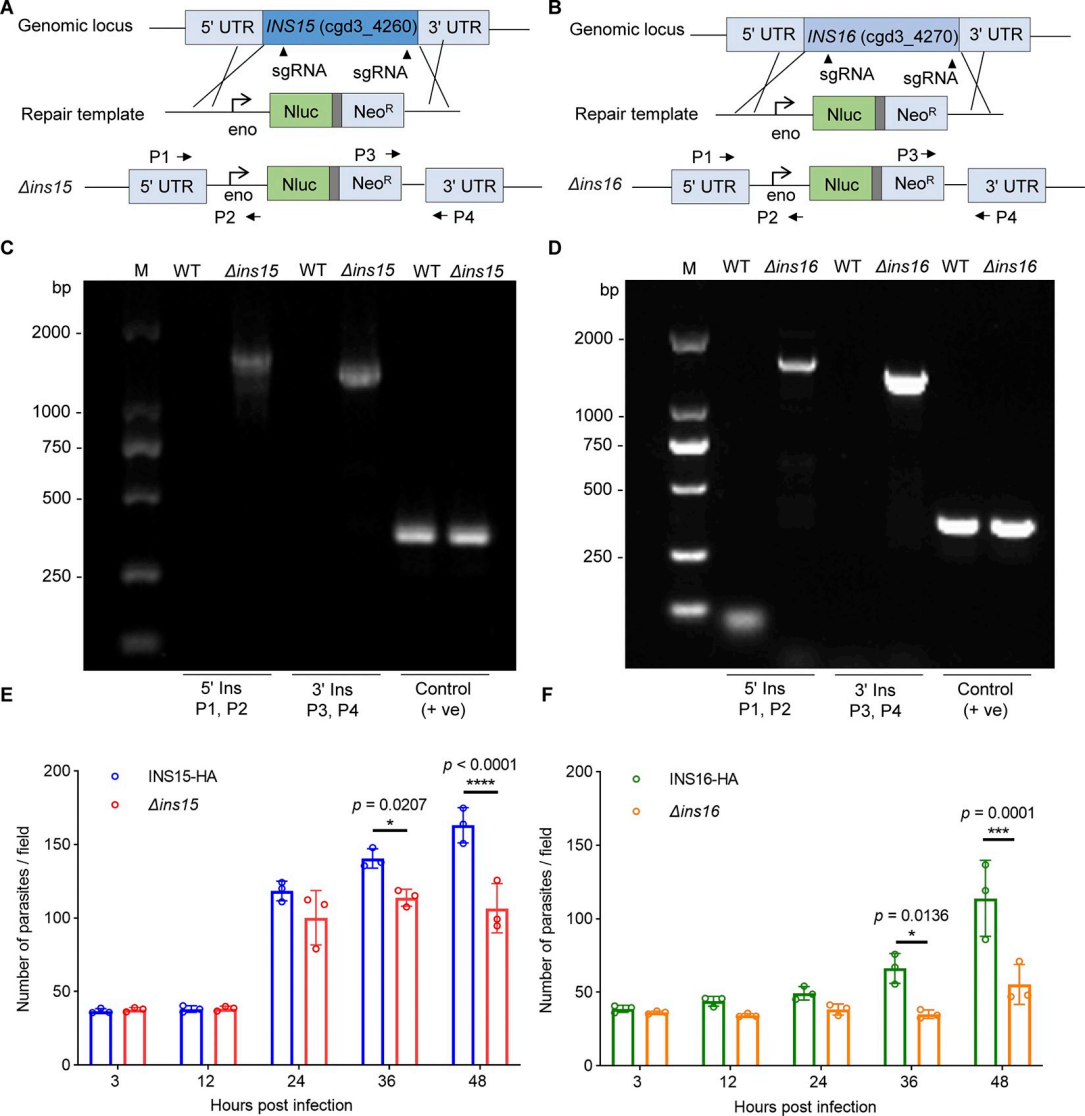
INS15 and INS16 are not essential for parasite survival. (A-B) Schematic shows the knockout strategy of *INS15* and *INS16*. The top line shows the native locus of *INS15* or *INS16*, the middle line shows the repair template, and the bottom line shows the modified locus. sgRNA, single guide RNA; Nluc, nano-luciferase; Neo^R^, neomycin resistance marker; Eno, enolase promoter. P1, P2, P3, and P4 are primers used to verify genomic integration events. (C-D) Diagnosis PCR of the *Δins15* and *Δins16* locus. Fecal genomic DNA from WT, *Δins15* and *Δins16* parasites were used as template, and primers for checking 5’ insertion (5’ Ins), 3’ insertion (3’ Ins), and the 3’ UTR sequence of INS16 as a positive control (+ve) are indicated. (E-F) Growth patterns of *Δins15* and INS15-HA strain (C) or *Δins16* and INS16-HA strains (D) in HCT-8 cell cultures. Data from three independent experiments, bars are standard deviations; *p* = exact values shown as determined with two-way ANOVA corrected for multiple comparisons according to Sidak’s method.

To investigate the role of INS15 and INS16 in *C*. *parvum*, we attempted to deplete the *INS15* and *INS16* gene using CRISPR/Cas9. We used CRISPR/Cas9 to generate a double-stranded DNA break in the *INS15* and *INS16* gene by two specific guide RNA sequences, and replacing endogenous gene locus with a Nluc-Neo^R^ selection cassette (Fig 3A and 3B). Surprisingly, we easily obtained the knockout strains of each gene. Mice were able to shed transgenic oocysts and subsequent PCR amplification of genomic DNA confirmed that knockout strains had corrected 5’ and 3’ integration (Fig 3C and 3D). *INS15* and *INS16* were successfully knocked out in *C*. *parvum*, suggesting that they are not essential for parasite survival.

### INS15 regulates parasite growth *in vitro* and *in vivo*

*Cryptosporidium* undergoes asexual reproduction from 0 to 24 hours post infection (hpi) and then enters sexual reproduction after 36 hpi in HCT-8 cells [26]. To further assess the effect of the *INS15* and *INS16* genes on growth and development of *Cryptosporidium*, we compared the proliferation of the knockout strain with that of tagging strain in HCT-8 cells. During the asexual reproduction stage (i.e. 3 hpi, 12 hpi and 24 hpi), lacking INS15 or INS16 did not affect *Cryptosporidium* invasion and proliferation. In contrast, depletion of INS15 or INS16 resulted in a significant decrease in the proliferation rate during the sexual reproduction stage (i.e. 36 hpi and 48 hpi) (Fig 3E and 3F), suggesting that INS15 may be involved in the development of male and female gametes and INS16 may be only involved the development female gametes *in vitro*.

**Fig 4:**
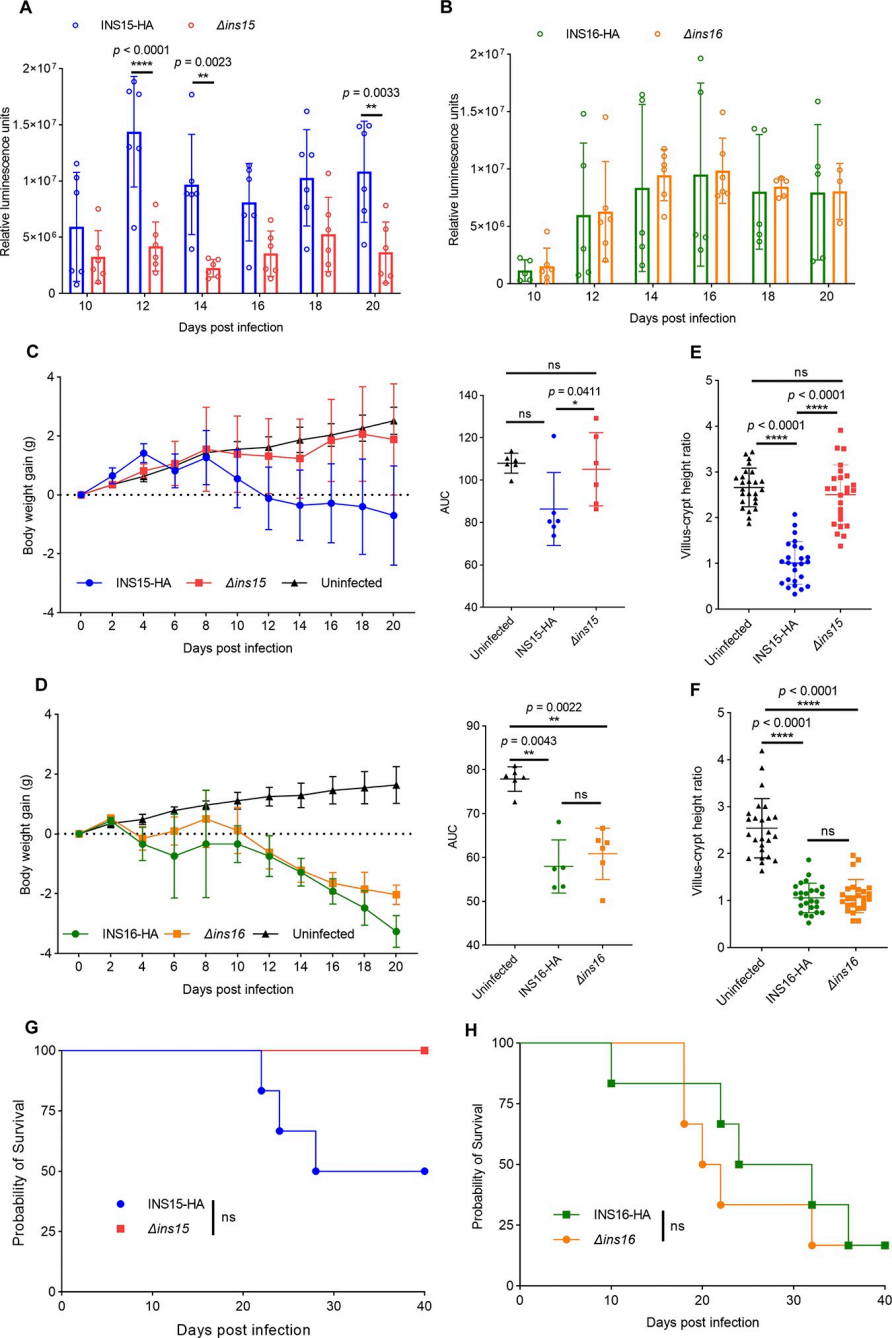
Depletion of INS15 reduces parasite virulence in infected GKO mice. (A-B) Fecal luminescence of GKO mice infected with *Δins15* or INS15-HA strain (A) and *Δins16* or INS16-HA strain (B). Each strain represents as mean ± SD (n = 6 from one experiment, each mouse was housed individually). *p* = exact values shown as determined with two-way ANOVA corrected for multiple comparisons according to Sidak’s method. (C-D) Measurement of body weight gain (left) and area under the curve (AUC) (right) of GKO mice orally infected with INS15-HA, *Δins15*, INS16-HA or *Δins16* oocysts. Body weight gain (g) normalized to the day before infection, n = 6 mice per group. *p* = exact values shown as determined with two-tailed Mann-Whitney U tests. (E-F) Measurement of villus length and crypt depth ratio for villi from mice infected INS15-HA, *Δins15*, INS16-HA or *Δins16*. Data from two independent experiments, n = 25; *p* = exact values shown as determined with two-tailed Mann-Whitney U tests. (G-H) Survival curve analysis for GKO mice infected with INS15-HA, *Δins15*, INS16-HA or *Δins16*. Statistical significance was tested using the log-rank Mantel-Cox test.

To further investigate the effects of INS15 and INS16 on *Cryptosporidium* growth *in vivo*, GKO mice were infected with tagging or knockout oocysts of each transgenic strain and the intensity of oocyst shedding was compared based luminescence values. The results showed that fecal luminescence values from mice infected with the *Δins15* strain decreased significantly with a 3.4-fold decrease starting at 12 dpi (Figs 4A and S2A). In contrast, luciferase activity in mice infected with the *Δins16* strain was not shown significantly differences during infection (Figs 4B and S2B). These results suggest that only deletion of INS15 can affect oocyst shedding *in vivo*.

### Depletion of INS15 reduces pathogenicity of *C*. *parvum*

We further compared the effects of different transgenic strains on host pathogenicity. The results showed that the body weight of mice infected with the INS15-HA strain decreased at 8 dpi, while that of mice infected with the *Δins15* strain maintained an increasing trend (Fig 4C). In contrast, the body weights of both INS16-HA and *Δins16* strain infected mice showed a significant decreasing trend from 8 dpi and there was no significant difference between INS16-HA infected mice and *Δins16* infected mice (Fig 4D).

To investigate the pathological changes of mice infected with various transgenic strains, the histological analysis was used to measure the villus-crypto height of the small intestine at 12 dpi. The results showed that the ratio of villus length to crypt depth in mice infected with the *Δins15* strains was significantly higher than that in mice infected with the INS15-HA strain (Fig 4D), indicating that deletion of INS15 significantly attenuated parasite damage to the intestinal epithelium. In contrast, INS16 deletion did not relieve parasite damage to the host intestine (Fig 4F). In addition, the survival curve of infected mice showed that GKO mice infected with the *Δins15* strain did not cause mortality in mice during the 40 days of infection, while mice infected with the INS15-HA strain resulted in 50% mortality (Fig 4G). In contrast, there was no difference of the survival curve between the mice infected with *Δins16* strain and the mice infected with INS16-HA strain (Fig 4H). Taken together, these results suggested that ablation of INS15 decreases oocysts shedding in GKO mice and improves mouse survival after parasite infection.

## Discussion

*Cryptosporidium parvum* INS15 and INS16 share 90% nucleotide sequence similarity and play distinct roles in *Cryptosporidium* infection of the host. Endogenous gene tagging showed that the expression patterns of INS15 and INS16 are very similar during the developmental stages of *Cryptosporidium*, and the difference between them is that INS16 is not expressed in the male gamont. Deletions of INS15 and INS16 individually significantly affected the sexual reproductive stages of *C*. *parvum*, while deletion of INS15 resulted in a significant reduction in oocyst shedding and attenuated damage to the host intestinal epithelium. These studies suggest that despite high sequence similarity, there are differences in function and that INS15 is involved in the sexual reproductive phase of the life cycle and correlates with pathogenicity.

INS15 and INS16 sequences share high sequence identity but have distinct expression profiles. Insulinases are proteases containing the M16 structural domain and are widely distributed in prokaryotes and eukaryotes. *C*. *parvum* genome encodes an extended family of 22 INS proteins, of which INS1, INS4, and INS15 have four complete functional domains, while other protein members have only 1–3 conserved structural domains [25]. In the present study, endogenous gene tagging results indicated that INS15 is more widely expressed than INS16. INS15 is expressed during all developmental stages. In contrast, INS16 is not expressed at the male gamete stage. This is consistent with previous studies using polyclonal antibodies against INS16 [18]. In addition, immunoelectron microscopy results showed that INS15 and INS16 were abundantly expressed on female gametes, with INS16 accumulating in large numbers mainly on amyloid granules, while INS15 aggregated on subcellular structures resembling dense granule-like structures. This suggests that INS15 and INS16 may play different roles in the sexual reproductive stages of *C*. *parvum*.

INS15 and INS16 are involved in the sexual reproduction stage of *C*. *parvum*. In the HCT-8 cellular infection model, *Cryptosporidium* entered the sexual reproduction stage at 36 hpi, forming male and female gametes sequentially, but the female gametes could not complete the fertilization process to form oocysts [26]. In this study, the results of intracellular proliferation of *Δins15* and *Δins16* strains showed that it was significantly reduced at 36 hpi, suggesting that deletion of these two genes affects the sexual reproduction stage of *Cryptosporidium*. Since INS15 is not expressed in the male gamete stage and may affect the formation of female gametes and the proliferation of *Cryptosporidium in vitro*. Recent studies of sexual reproduction in *Cryptosporidium* have identified several maker molecules that are specifically expressed during the male and female gamete stages, among which the apetala 2 transcription factor (AP2) and INS1 have been shown to be characteristically expressed during the female gamete stage of *Cryptosporidium*, and INS1 is involved in the process of oocyst formation [19,27,28]. CDPK5 is specifically expressed in the male gamete stage, and CDPK5 directly influences the pathogenicity of the parasite to the host [29].

INS15 affects parasite burden in mice, and it is associated with the pathogenicity of *C*. *parvum*. Several protein families, such as mucins, transporters (ABC and ATPase3), and cysteine proteases, have been associated with *Cryptosporidium* host pathogenicity [30]. Due to the lack of methods to manipulate the *Cryptosporidium* genome, it has not been possible to knock out or suppress genes and confirm that their absence or inactivation reduces the severity of cryptosporidiosis [30]. Recent advances in the genetic manipulation of *Cryptosporidium* have allowed the identification of a relationship with *C*. *parvum* pathogenicity [31]. Previous findings show that loss of CDPK5 reduces the number of male gametes, oocyst production and shedding in infected mice, resulting in an overall lower intestinal burden and virulence observed in IFN-γ KO mice [29]. Our results clearly show that deletion of the *INS15* gene significantly reduces the growth of the sexual reproductive stage of *C*. *parvum in vivo*, and mice infected with the *Δins15* strain showed a significant reduction in infection intensity and improved survival of infected mice, whereas deletion of INS16 was not associated with pathogenicity. The above studies demonstrate that the factors affecting the pathogenicity of *Cryptosporidium* are related to a variety of factors in which genes associated with the sexual reproduction stage play an important role. Our results indicate that INS15 is involved in the process of oocyst formation in *C*. *parvum*, which is associated with pathogenicity. We acknowledge that our study does not clearly define the biological functions of INS15 and INS16. Therefore, further biological investigations into the functions of INS15 and INS16 can be conducted.

## Supporting information

S1 Fig Immunoelectron microscopy of INS15-HA and INS16-HA parasites at in trophozoite and meront stages. Few gold particles were scattered during trophozoite and meront stages as indicated by the red arrows, it is impossible to determine their expression in subcellular organelles. Scale bars = 0.5 μm.
(TIF)

S2 Fig Oocyst shedding from GKO mice infected with transgenic strains based on luminescence values. Measurement of luminescence values (left) and area under the curve (AUC) (right) of GKO mice orally infected with transgenic strains. (A) Oocyst shedding from GKO mice infected with INS15-HA or *Δins15*. (B) Oocyst shedding from GKO mice infected with INS16-HA or *Δins16*.
(TIF)

S1 Table Primers used in this study. (XLSX)
