# Supplementary figures and images for: Involvement of INS15 in the development and pathogenicity of the zoonotic pathogen Cryptosporidium parvum
Source: PLoS Negl Trop Dis. 2024 Oct 3;18(10):e0012569. doi: 10.1371/journal.pntd.0012569 (PMC11478815; doi:10.1371/journal.pntd.0012569)

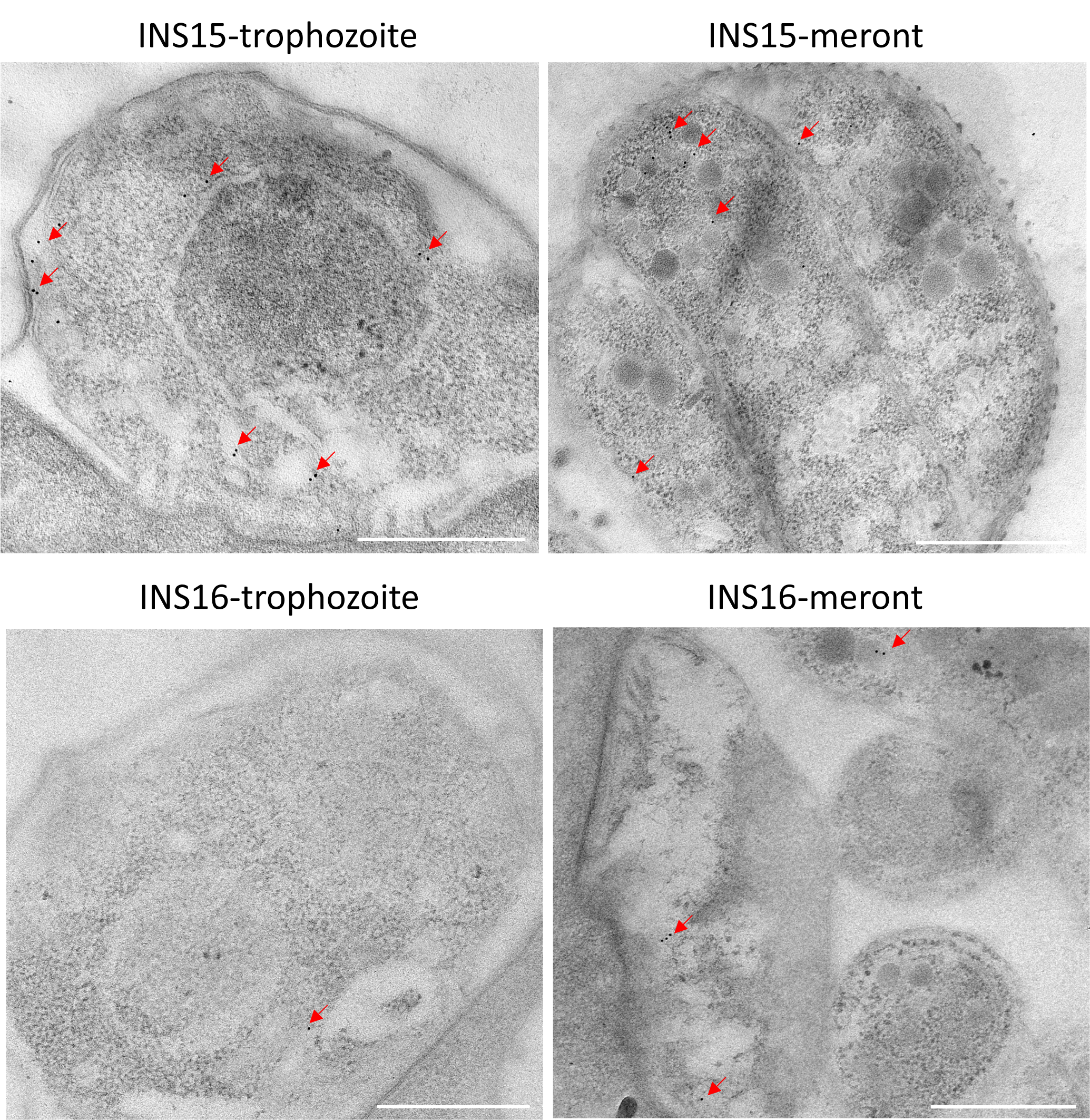

Supplement: S1 Fig — Few gold particles were scattered during trophozoite and meront stages as indicated by the red arrows, it is impossible to determine their expression in subcellular organelles. Scale bars = 0.5 μm. (TIF) [file pntd.0012569.s001.tif]

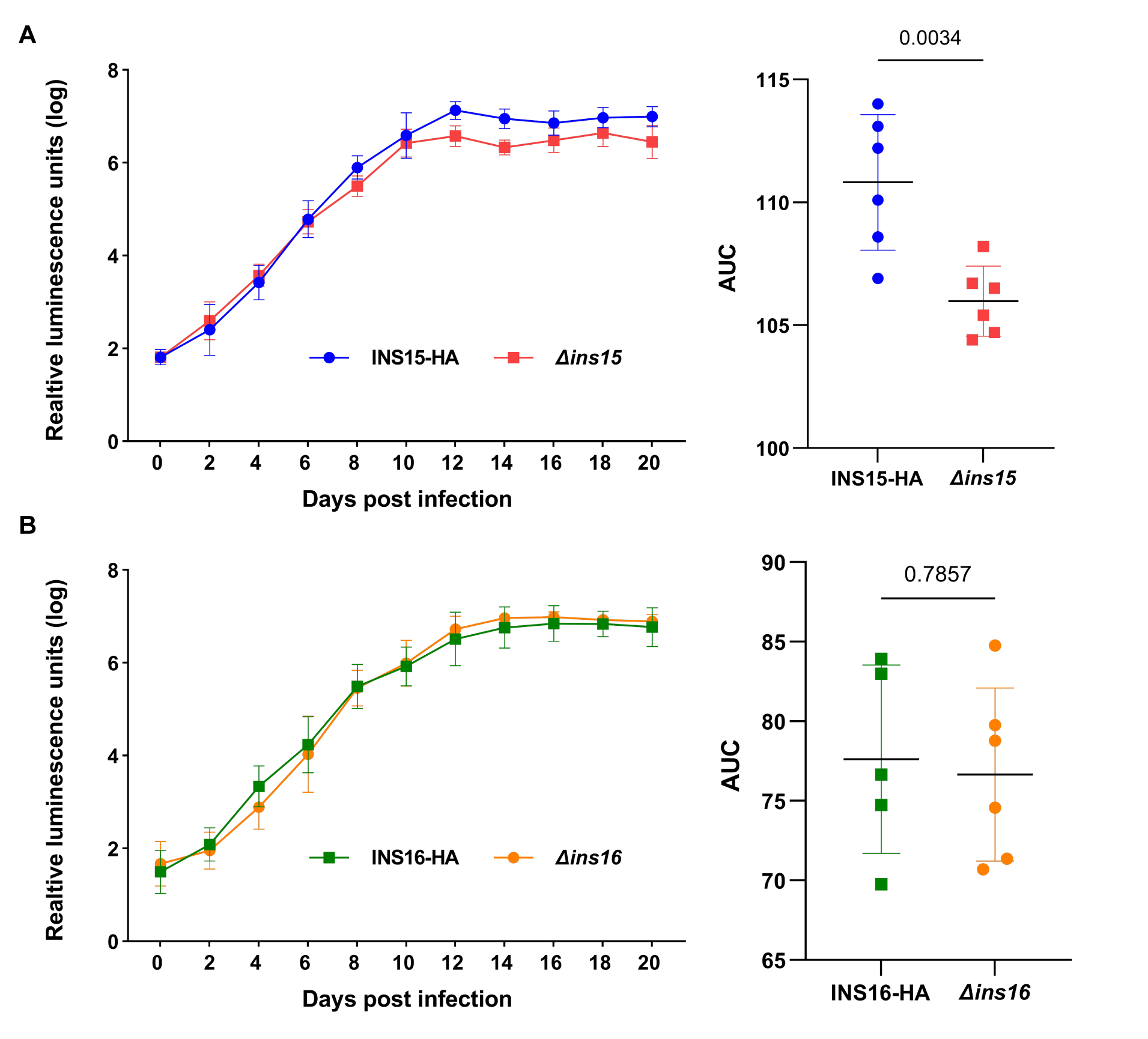

Supplement: S2 Fig — Measurement of luminescence values (left) and area under the curve (AUC) (right) of GKO mice orally infected with transgenic strains. (A) Oocyst shedding from GKO mice infected with INS15-HA or Δins15. (B) Oocyst shedding from GKO mice infected with INS16-HA or Δins16. (TIF) [file pntd.0012569.s002.tif]
